# Longitudinal changes in leukocyte telomere length and mortality in elderly Swedish men

**DOI:** 10.18632/aging.101611

**Published:** 2018-10-29

**Authors:** Xiaotian Yuan, Magnus Kronström, Mai-Lis Hellenius, Tommy Cederholm, Dawei Xu, Per Sjögren

**Affiliations:** 1 Department of Medicine, Division of Hematology, and Center for Molecular Medicine, Karolinska Institutet and Karolinska University Hospital Solna, SE-171 76 Stockholm, Sweden; 2 Department of Public Health and Caring Sciences, Unit of Clinical Nutrition and Metabolism, Uppsala University, Uppsala, Sweden; 3 Karolinska Institutet, Department of Medicine, Karolinska University Hospital, Solna, SE-171 76 Stockholm, Sweden; * Equal contribution

**Keywords:** telomere length, longitudinal changes, mortality, cause-specific mortality, cohort study

## Abstract

Telomere length (TL) is considered an indicator of aging and age-related diseases, but longitudinal studies on TL changes and mortality are few. We therefore analyzed TL and longitudinal changes in TL in relation to all-cause, cardiovascular, and cancer mortality in 247 elderly Swedish men. TL was determined by the qPCR method at ages 71 and 81 and subsequent mortality cases were identified from the Swedish cause-of-death registry. Cox proportional hazard ratios were calculated during a mean follow-up of 7.4 years, during which 178 deaths occurred. Short telomeres at baseline was strongly associated with mortality risks, with a 40 to 70% increased risk of all-cause mortality, and a 2-fold increased risk of cancer mortality. Longitudinal changes in TL revealed shortening in 83% of individuals, whilst 10% extended their telomeres. TL attrition did not predict all-cause or cancer mortality, but we found a 60% decreased risk for cardiovascular mortality in those who shortened their telomeres. Our data show an increased risk of mortality in individuals with short baseline telomeres, but no relations to all-cause, and cancer mortality for changes in TL. Intriguingly, our data indicate lower risk of cardiovascular mortality with shortening of telomeres. The latter should be interpreted cautiously.

## Introduction

Telomeres are structures at the end regions of chromosomes in eukaryotic organisms. They consist of repeated, species specific, sequences of hexanucleotides, which partly serve to prevent unlimited cell replication and to protect the cell from genome instability [1]. At birth telomere length (TL) is hereditary and varies significantly between individuals [2]. During life, there is a balance between telomere shortening and telomere extension, but TL is typically shortened with each cell cycle (due to the end-replication problem) yielding shorter telomeres with aging [3]. Environmental and lifestyle factors have been identified to influence telomere shortening [4-6], but their role in TL dynamics remains elusive.

TL has been proposed as a promising biological clock of aging [7], and strong relationships to age-related diseases have been described [8,9]. Many studies have also reported shorter telomeres to be associated with increased mortality [10-21], whilst others have not [22-27]. There are few longitudinal studies on TL and cause-specific mortality in the literature. To our knowledge, there are only three studies based on healthy individuals that have assessed longitudinal changes in TL in relation to mortality. Two of these studies found no relationship between telomere attrition and mortality [22,25], and the third study found a relationship only to cardiovascular mortality [13]. The general absence of relationship in those studies may be explained by the age of study participants and short follow-up. The mean ages at baseline in the first two studies were 90 and 45 years, respectively, and changes in TL might be a poor predictor of mortality at those ages. The third study, which found a relationship to cardiovascular mortality but not to total mortality, had a limited follow-up period of only 2.5 years. Study population and sample size, together with e.g. measurement techniques, represents likely caveats that may hamper possibilities to capture TL attrition as a predictor of mortality.

We hypothesized that short telomeres and especially shortening of telomeres could be related to all-cause and cause-specific mortality in a population-based study of elderly individuals. Therefore, data from the ULSAM (Uppsala Longitudinal Study of Adult Men) cohort was used to assess whether TL and longitudinal changes in TL were related to all-cause, cardiovascular, and cancer mortality in elderly Swedish men.

## RESULTS

Baseline characteristics of our 247 participants examined at age 71 and 81 are depicted in Table 1, revealing a rather healthy set of individuals being only slightly obese, with few smokers and being relatively physically active. During follow-up, the study group became less active and smoking habits improved, but they remained relatively weight stable. At age 81, a majority of the participants had pharmacological treatment and most of them had received cardiovascular (49%) or cancer (21%) diagnosis. On average, TL decreased by 30% in the study group, 83% displayed TL attrition and 10% extended their telomeres, defined as more than 15% loss or extension in TL between examinations. Furthermore, a strong relationship between baseline TL and loss in TL was found (β=0.76, p<0.0001 for absolute values); i.e. longer baseline telomeres was related to a higher loss in TL. During the mean follow-up time of 7.4 years, using the 81-year-examination as starting-point, 178 (72%) deaths occurred, of which 65 cases (37%) were due to cardiovascular disease and 61 (34%) were due to cancer.

**Table 1:** Characteristics of Study Subjects Participating in the Uppsala Longitudinal Study of Adult Men (ULSAM) Cohort in 1991 and 2002, Uppsala, Sweden.

	**Examination at age 71**			**Examination at age 81**	
	n			n	
Age (years)	247	70.8 (0.7)		247	81.3 (0.8)
Body mass index (kg/m^2^)	246	26.2 (2.9)		246	26.2 (3.2)
Alcohol intake (g/d)	241	7.7 (7.5)		144	8.7 (9.2)
Current smokers, n (%)	244	33 (13)		243	16 (6)
Physical activity, n (%)	240			239	
Sedentary		5 (2)			29 (12)
Moderate		82 (34)			94 (39)
Regular		136 (57)			104 (44)
Athletic		17 (7)			12 (5)
Education, n (%)	247			247	
6-7 years		107 (43)			107 (43)
8-13 years		84 (34)			84 (34)
>13 years		56 (23)			56 (23)
Antihypertensive medication, n (%)	241	75 (31)		231	126 (55)
Antidiabetic medication, n (%)	241	9 (4)		231	24 (10)
Lipid lowering medication, n (%)	241	28 (12)		231	56 (24)
Type-2-Diabetes, n (%)	243	9 (4)		245	25 (10)
Cardiovascular diagnosis before examination, n (%)	247	70 (28)		247	120 (49)
Cancer diagnosis before examination, n (%)	247	15 (6)		247	51 (21)
Telomere length, t/s ratio	247	1.50 (0.52)		247	0.88 (0.38)
Telomere length, % change	247	NA		247	-30 (60)
Telomere length attrition, n (%)	247	NA		247	206 (83)

Values are mean (SD) if not otherwise stated

### All-cause mortality

Baseline TL from the 71-year-examination was strongly and inversely related to all-cause mortality (Table 2). For example, the crude hazard ratio (HR, 95% CI) for all-cause mortality in low vs. high tertile of TL was 1.66 (1.15-2.39), which remained essentially unchanged when adjusted for possible confounders (fully adjusted HR, 1.72 (1.17-2.52)). The inverse relationship between TL and all-cause mortality was also seen when TL was applied as a continuous variable with a fully adjusted HR of 1.71 (1.27-2.29). Note that the continuous measure was multiplied with -1 to reflect increased risk of mortality with shorter telomere length. Similar associations, yet weaker and non-significant, were found when baseline TL from the 81-year-examination was used (e.g. a fully adjusted HR of 1.44 (0.92-2.24) for low vs. high tertile of TL). As also depicted in Table 2, there were no clear association between TL attrition and all-cause mortality. In crude analyses, individuals who shortened their telomeres had a HR of 0.66 (0.45-0.96), but this relation was completely abolished when adjusted for covariates (including baseline TL). Moreover, we found no relation between TL attrition and all-cause mortality in our subgroup analysis, performed only in individuals with short telomeres at baseline and comparing those who displayed TL attrition with those who did not.

**Table 2:** Hazard ratios (95% CI) for baseline and longitudinal changes in Telomere length (TL) in relation to all-cause mortality.

	**Number of deaths**		**Crude**			**Minimally adjusted^b, c^**			**Fully adjusted^d^**	
			**HR**	**95% CI**		**HR**	**95% CI**		**HR**	**95% CI**
**TL at age 71**										
TL (continuous)^a^	178		1.65	1.24, 2.18		1.62	1.22, 2.16		1.71	1.27, 2.29
TL tertiles										
High	52		1.00	Referent		1.00	Referent		1.00	Referent
Medium	59		1.21	0.80, 1.76		1.31	0.88, 1.96		1.29	0.85, 1.94
Low	67		1.66	1.15, 2.39		1.62	1.11, 2.37		1.72	1.17, 2.52
										
**TL at age 81**										
TL (continuous)^a^	178		1.32	0.84, 2.07		1.27	0.78, 2.05		1.42	0.86, 2.34
TL tertiles										
High	57		1.00	Referent		1.00	Referent		1.00	Referent
Medium	59		1.07	0.74, 1.54		0.98	0.66, 1.46		0.92	0.60, 1.41
Low	62		1.34	0.93, 1.92		1.30	0.86, 1.95		1.44	0.92, 2.24
										
**Longitudinal change in TL**										
TL maintained/lengthened (n=41)	34		1.00	Referent		1.00	Referent		1.00	Referent
TL attrition (n=206)	144		0.66	0.45, 0.96		0.75	0.45, 1.24		0.83	0.49, 1.42
										
Short baseline TL with no TL attrition (n=32)	27		1.00	Referent		1.00	Referent		1.00	Referent
Short baseline TL with TL attrition (n=50)	40		0.80	0.49, 1.31		0.79	0.37, 1.70		1.01	0.40, 2.56

^a^ continuous measures were multiplied with -1 to reflect increased risk of mortality with shorter telomere length

^b^ adjusted for age, BMI, smoking, alcohol intake, physical activity and education; ^c^ for longitudinal changes also adjusted for baseline TL (at age 71). ^d^ all covariates in the minimally adjusted model + self-reported T2D, CVD diagnosis before baseline, cancer diagnosis before baseline, and pharmacological treatment of hypertension, T2D or dyslipidemia.

### Cardiovascular mortality

Baseline TL from the 71-year-examination was inversely related to cardiovascular mortality (Table 3) with a fully adjusted HR of 1.76 (1.10-2.82) for the (inverted) continuous measure of TL. The corresponding relations for tertiles revealed a non-significant fully adjusted HR of 1.75 (0.94-3.28) among individuals with the shortest telomeres compared to those with the longest telomeres. We found no indication of relationships between TL and cardiovascular mortality when applying TL-data from the 81-year-examination (Table 3). However, we found a significantly lower HR of cardiovascular mortality among those who shortened their telomeres. For example, the fully adjusted HR in the TL attrition group was 0.42 (0.19-0.89) compared to those who did not shorten their telomeres (Table 3). This relation, though, was not found in the subgroup of individuals with short telomeres at baseline, with a fully adjusted HR of 1.10 (0.28-4.39) among those who displayed TL attrition as compared to those who did not.

**Table 3:** Hazard ratios (95% CI) for baseline and longitudinal changes in Telomere length (TL) in relation to cardiovascular mortality.

	**Number of deaths**		**Crude**			**Minimally adjusted^b, c^**			**Fully adjusted^d^**	
			**HR**	**95% CI**		**HR**	**95% CI**		**HR**	**95% CI**
**TL at age 71**										
TL (continuous)^a^	65		1.66	1.06, 2.62		1.64	1.03, 2.60		1.76	1.10, 2.82
TL tertiles										
High	18		1.00	Referent		1.00	Referent		1.00	Referent
Medium	21		1.26	0.67, 2.37		1.26	0.63,2.53		1.17	0.58, 2.37
Low	26		1.73	0.95, 3.16		1.65	0.89, 3.06		1.75	0.94, 3.28
										
**TL at age 81**										
TL (continuous)^a^	65		0.80	0.44, 1.45		0.84	0.45, 1.59		0.95	0.51, 1.74
TL tertiles										
High	27		1.00	Referent		1.00	Referent		1.00	Referent
Medium	18		0.68	0.37, 1.23		0.64	0.34, 1.24		0.55	0.27, 1.10
Low	20		0.87	0.49, 1.56		0.93	0.49, 1.75		1.03	0.53, 2.00
										
**Longitudinal change in TL**										
TL maintained/lengthened (n=41)	19		1.00	Referent		1.00	Referent		1.00	Referent
TL attrition (n=206)	46		0.42	0.24, 0.71		0.40	0.19, 0.85		0.42	0.19, 0.89
										
Short baseline TL with no TL attrition (n=32)	14		1.00	Referent		1.00	Referent		1.00	Referent
Short baseline TL with TL attrition (n=50)	12		0.50	0.23, 1.08		0.72	0.21, 2.45		1.10	0.28, 4.39

^a^ continuous measures were multiplied with -1 to reflect increased risk of mortality with shorter telomere length.

^b^ adjusted for age, BMI, smoking, alcohol intake, physical activity and education; ^c^ for longitudinal changes, also adjusted for baseline TL (at age 71). ^d^ all covariates in the minimally adjusted model + self-reported T2D, CVD diagnosis before baseline, and pharmacological treatment of hypertension, T2D or dyslipidemia.

### Cancer mortality

Baseline TL from the 71-year-examination was inversely related to cancer mortality (Table 4) with a fully adjusted HR of 1.99 (1.21-3.28) for the (inverted) continuous measure of TL. The corresponding relations for tertiles revealed a fully adjusted HR of 2.13 (1.13-4.01) among individuals with the shortest telomeres as compared to those with the longest telomeres. When baseline measures of TL at the 81-year-examination were applied we found similar numerical associations, but with wider confidence intervals. The fully adjusted HRs were 1.93 (0.78-4.78) for TL as a continuous (inverted) variable, and 2.53 (1.14-5.63) for low vs. high tertile of TL. As also depicted in Table 4, there were no clear associations between TL attrition and cancer mortality. Individuals who shortened their telomeres had a fully adjusted HR of 1.15 (0.46-2.87), compared to those who maintained/lengthened their telomeres and further no relationship between TL attrition and cancer mortality was found among those with the shortest telomeres as baseline.

**Table 4:** Hazard ratios (95% CI) for baseline and longitudinal changes in Telomere length (TL) in relation to cancer mortality.

	**Number of deaths**		**Crude**			**Minimally adjusted^b, c^**			**Fully adjusted^d^**	
			**HR**	**95% CI**		**HR**	**95% CI**		**HR**	**95% CI**
**TL at age 71**										
TL (continuous)^a^	61		1.80	1.13, 2.87		1.81	1.12, 2.94		1.99	1.21, 3.28
TL tertiles										
High	17		1.00	Referent		1.00	Referent		1.00	Referent
Medium	17		1.09	0.56, 2.14		0.98	0.48, 2.00		0.88	0.42, 1.84
Low	27		1.93	1.05, 3.55		1.92	1.03, 3.58		2.13	1.13, 4.01
										
**TL at age 81**										
TL (continuous)^a^	61		1.31	0.62, 2.79		1.30	0.56, 3.02		1.93	0.78, 4.78
TL tertiles										
High	17		1.00	Referent		1.00	Referent		1.00	Referent
Medium	18		1.07	0.55, 2.08		1.00	0.47, 2.14		1.16	0.51, 2.63
Low	26		1.78	0.97, 3.28		1.82	0.91, 3.63		2.53	1.14, 5.63
										
**Longitudinal change in TL**										
TL maintained/lengthened (n=41)	12		1.00	Referent		1.00	Referent		1.00	Referent
TL attrition (n=206)	49		0.69	0.37, 1.30		0.97	0.41, 2.29		1.15	0.46, 2.87
										
Short baseline TL with no TL attrition (n=32)	10		1.00	Referent		1.00	Referent		1.00	Referent
Short baseline TL with TL attrition (n=50)	17		0.97	0.45, 2.13		1.01	0.31, 3.28		1.33	0.29, 6.03

^a^ continuous measures were multiplied with -1 to reflect increased risk of mortality with shorter telomere length.

^b^ adjusted for age, BMI, smoking, alcohol intake, physical activity and education; ^c^ for longitudinal changes also adjusted for baseline TL (at age 71). ^d^ all covariates in the minimally adjusted model + self-reported T2D, cancer diagnosis before baseline, and pharmacological treatment of hypertension, T2D or dyslipidemia.

## DISCUSSION

In the present study we investigated whether baseline TL at both age 71 and 81, as well as longitudinal changes in TL, were related to mortality in elderly men. In our population, baseline TL was strongly associated with mortality, with a 40 to 70% increased risk of all-cause mortality, and over 2-fold increased risk of cancer mortality among individuals with short telomeres. The corresponding risk relations for cardiovascular mortality were more inconsistent, indicating that short telomeres at age 71, but not 81, may enhance the risk of future cardiovascular mortality. For longitudinal changes, TL attrition was apparent in an absolute majority of our individuals (83%), but 10% actually extended their telomeres. Extension of telomeres have previously been described in the literature [28], although some claim that this is an effect of measurement error [29]. However, we find measurement error an unlikely explanation to TL extension in our study group considering the long time-span between examinations and a limited CV in our qPCR assays. Our risk analyses for longitudinal changes revealed that TL attrition did not predict all-cause or cancer mortality, but we found a 60% decreased risk for cardiovascular mortality in those who shortened their telomeres. This unexpected finding remained robust after controlling for influential confounders such as baseline TL, pronounced cardiovascular risk factors, and previous cardiovascular diagnosis.

Overall, our data indicate increased risk of mortality with short telomeres at baseline, a relationship that was especially strong when using TL-data derived from the 71-year-examination. This is in line with most previous studies [10-21], but not all [22-27], describing a negative relationship between baseline TL and mortality. Previous studies also indicate that this relation becomes weaker with increasing age [30]. The latter is coherent with our observations, in which risk relations were strong when baseline was set to 71 years, but not when using the 81-year-examination. A weakened relationship with increasing age might be anticipated considering the loss in TL that occurs in most individuals, and especially in those who start with long telomeres. The distribution of TL in our individuals at age 71 was wider than at age 81, indicating that our population became more homogenic in terms of TL as they grew older. This would in turn limit the possibilities to detect risk associations. Still, we found a pronounced relation between short telomeres and future cancer mortality, independent of choice of baseline. This relation became especially strong after adjusting for potential confounders, such as prevalent cancer disease. Previous studies on TL and cancer mortality reveal conflicting results [9], and whether TL has a causative role remains to be established. It is likely that the relation may differ for different cancer forms and further being highly dependent on follow-up time, age of study participants, and possibilities to control for influential confounders. Today, most attention within this field is given to the role of telomerase, an enzyme that is responsible for elongating telomeres. The ability to maintain an adequate TL, by upregulating telomerase activity, has been proposed as a prerequisite for tumor development, but current data suggest a more complex scenario [9].

We further analyzed whether longitudinal changes in TL could predict all-cause or cause-specific mortality. This was also tested in a sub-group of individuals who had short telomeres at baseline and displayed TL attrition between the two examinations. The rationale for the latter approach relates to a study by Epel et al, in which extremely high 12-year mortality was found among men with short TL at baseline, when comparing those who experienced TL shortening with those who did not [13]. Interestingly, we were not able to replicate this, with overall null findings. TL attrition in this sub-group was neither related to all-cause, cardiovascular nor cancer mortality. The discrepancy between these two studies is hard to explain, but supposedly statistical power could play a role. Although the size of our sub-group was limited (n=82) it was higher than in the aforementioned study, which should limit the risk of chance findings. However, corresponding analyses in the whole study group, comparing those who experienced TL attrition (n=206) with those who did not (n=41), revealed some interesting results. After controlling for essential confounders, TL attrition did neither predict all-cause nor cancer mortality. However, individuals who experienced TL attrition had a lower risk of future cardiovascular mortality than those who maintained or lengthened their telomeres. This risk relation remained after controlling for influential confounders and indicated a 60% risk reduction of dying from cardiovascular disease. The notion that shortening of telomeres would protect against cardiovascular mortality is novel and intriguing, and available data on longitudinal changes in TL are not coherent with our findings. On the contrary, a recent study on patients with stable coronary heart disease found decreased mortality in patient who lengthened their telomeres [31]. Moreover, a newly published study on lifelong changes in TL found a more pronounced TL shortening among CVD patients compared with non-CVD patients [32]. Finally, a study on breast cancer survivors found increased overall mortality risk (cardiovascular mortality was not assessed) in those who shortened their telomeres [33]. It is noteworthy, though, that the two aforementioned mortality studies were based on individuals with an established diagnosis, and similar studies in healthy subjects do not convincingly show any increased risk of mortality with TL attrition. One study found an elevated risk of cardiovascular death among men who shortened their telomeres [13], but those results may have been affected by a short follow-up (2.5y) and missing covariates. Another study in the oldest old (~90y at baseline) could not detect any relation between TL attrition and future mortality [25], and one can argue that shortening of telomeres may be less important in this age group. A third study, with a large study sample, found no relation to total mortality for changes in TL over 10 years [22]. The time-span for assessing TL changes was similar to ours, but the participants were much younger (mean 45y at baseline). Still, those results are coherent with our data on all-cause mortality and it would have been interesting to know the corresponding results for cardiovascular mortality in that study.

Despite strengths of this study, such as well-characterized participants, valid follow-up time for the age group and minimal loss to follow-up, there were several limitations. First, we had a relatively limited sample size. This forced us to combine deaths from coronary heart disease and cerebrovascular disease, which may have affected our risk relations to cardiovascular mortality. It also prevented us from analyzing mortality from specific cancer forms, and since prostate cancer represents a major part of cancer deaths in our material (45%) we cannot exclude the possibility that those associations are driven by prostate cancer per se. Second, the possible influence of selection bias cannot be ignored since our participants at age 71 were healthy and survival up to the 81-year-examination was a prerequisite to be included in these analyzes. Third, technical issues such as precision of methodology and DNA quality may have influenced our results. However, we have no indication of poor DNA quality or exceptionally poor precision in our PCR method that would make our results unreliable. The length of our telomeres, though, may oscillate over time, as also suggested in the literature [28,34]. This would limit the possibilities of measuring longitudinal changes in TL with great precision. Finally, we cannot exclude the possibilities of residual confounding, and neither the possibility that some of our selected covariates may not be true confounders, which could yield distorted risk estimates. Still, risk estimates in our model with such covariates (i.e. the fully adjusted model), did not deviate substantially from the corresponding risk estimates in the minimally adjusted model.

In conclusion, our study confirms previous publications showing an overall shortening of TL length over time, and that TL extension occurs in a subset of individuals. Our data also indicate an increased risk of all-cause and cancer mortality, but not clearly so for cardiovascular mortality, in elderly men having short telomeres at baseline. Longitudinal changes were only related to cardiovascular mortality, with a pronounced risk reduction in those who shortened their telomeres. The latter result is intriguing and should be interpreted with some caution until additional studies may confirm such a relation.

## MATERIALS AND METHODS

### Participants and follow-up

This study is based on the Uppsala Longitudinal Study of Adult Men cohort (http://www.pubcare.uu.se/ULSAM/). All men born 1920-24 and living in Uppsala were at the age of 50 (1970-1974) invited to the study, and 82% (n=2322) agreed to participate. The population was followed over time, and re-examinations were performed at ages 60, 71, 77, 81, 88, and 92. For the present study, blood samples from participants who took part of examinations at both age 71 and 81 (n=257) were selected for TL analyses to determine changes in TL longitudinally. Ten individuals were excluded due to missing values for TL at either occasion, rendering 247 male participants for the present analyses. This study was conducted with ethical approval from the ethics committee at Uppsala University, and all participants gave informed consent.

A prerequisite for inclusion in this study was survival up until the examination at age 81, and thus follow-up was from the examination date at age 81 (performed April 2003 to April 2005) and to 31 December 2014, with a maximum of 11.7 y of follow-up and a mean of 7.4 y. The Swedish national registry recording for cause of death was used to define endpoints. The register includes all Swedish citizens, which minimizes loss to follow-up. The endpoints were defined a priori as cardiovascular mortality, i.e. ICD-9 codes 390-459, ICD-10 codes I00-I99; and cancer and total mortality.

### Baseline examination and covariates

Baseline investigations were performed under standardized conditions as described [35,36]. The investigation included a medical questionnaire and interview, blood sampling, as well as measurements of anthropometry and blood pressure. Fasting blood samples were drawn and circulating lipoproteins, triacylglycerol, cholesterol, glucose and insulin were determined as described [37]. Information on treatments for type-2-diabetes, hypertension and dyslipidemia, as well as status of smoking, physical activity and occupation was collected from the questionnaire. Smoking was defined as current smokers. Regular physical activity was defined as the reporting of regular or athletic leisure-time exercise habits according to four physical activity categories (sedentary, moderate, regular and athletic) [38]. Education was categorized as low (6-7y), medium (8-13y) and high (>13y). Alcohol intake in g/d was derived from a 7-d food record at age 71 and with a FFQ at age 81, as previously described [39].

### Telomere length quantification

DNA was extracted from blood samples by using either salting out DNA extraction method or QIAGEN kit (QIAGEN, Hilden, Germany). Quality was controlled by spectrophotometric analysis. Aliquots of purified DNA were provided to the laboratory of Dr. Dawei Xu, at Karolinska Institutet, Solna, Sweden, where the TL assay was performed. TL was measured using the quantitative polymerase chain reaction (PCR) method. Two ng of DNA were used for each PCR reaction. The primer sequences for human telomere (Tel 1b and Tel 2b) and β-globin (HBG3 and HBG4) were: Tel1b: 5’-CGGTTTGTTTGGGTTTGGGT-TTGGGTTTGGGTTTGGGTT-3’; Tel2b: 5’-GGCTTGCCTTACCCTTACCCTTACCC-TTACCCTTACCCT-3’; HBG3: 5’-TGTGCTGGCCCATCACTTTG-3’, and HBG4: 5’-ACCAGCCA-CCACTTTCTGATAGG-3’. T/HBG values were determined using the formula T/S = 2^-ΔCt^, where ΔCt = average Ct_telomere_ − average Ct_β-globin_. The T/S ratio was arbitrarily expressed as LTL.

To verify our TL data we re-analyzed DNA, extracted from two individuals at two separate time-points, using qPCR and Southern Blot. We used the TeloTAGGG™ Telomere Length Assay kit (Cat.#: 12209136001, Merck KGaA, Darmstadt, Germany) to determine TL by Southern Blot. Two μg of DNA were digested with RsaI/HinfI restriction enzymes with subsequent steps following the instructions from the manufacture. Signals were quantified using molecular weight markers as reference. As shown in Supplementary Figure 1, the length of the telomeres in these two individuals were overall consistent between methods, which also is consistent with the much larger validation study by Cawthon [40].

### Statistics

All analyses were performed using the STATA statistical package (Intercooled STATA 13.0 for Windows; Stata Corp, College Station; TX, USA) and significance level was set to 0.05. Cox proportional hazard regression analysis was used to calculate hazard ratios (HR) with two-tailed 95% confidence intervals [CI] for all-cause and cause-specific mortality, with time-on-study as time-scale. Censoring was done at the date of death or December 31, 2014. Proportional hazard assumptions were confirmed by Schoenfeldt’s test. We applied continuous and categorical variables of TL and changes in TL to our models. Categorical variables were generated as tertiles of baseline TL measures (using the upper tertile as reference group) and below or exceeding 15% change in TL for longitudinal changes. Thus, the latter categories were classified as TL-shortening (>15% loss in TL), TL-maintain (-15 to +15% change in TL) and TL-extension groups (>15% extension in TL) during follow-up; and we used a pooled variable combining TL-maintain with TL-extension as reference group. We also identified those individuals with short telomeres at baseline (low tertile) who displayed TL attrition over time; hazard ratios were calculated in this group using individuals with short telomeres at baseline but not displaying TL attrition as reference group.

To control for possible confounding, we applied a minimally adjusted model, including age (continuous), BMI (continuous), smoking (categorical), alcohol intake (continuous), physical activity (categorical) and education (categorical); additionally, the model for longitudinal changes in TL also included baseline TL. The fully adjusted model included all above variables in addition to treatments of hypertension, type-2-diabetes and dyslipidemia as well as self-reported type-2-diabetes, and previous diagnosis of cardiovascular disease or cancer. The two latter were both included when using all-cause mortality as outcome, whilst cause-specific mortality only included one or the other.

## Supplementary Material

Supplementary Figure
