# Supplementary material for: Longitudinal changes in leukocyte telomere length and mortality in elderly Swedish men
Source: Aging (Albany NY). 2018 Oct 29;10(10):3005–16. doi: 10.18632/aging.101611 (PMC6224259; doi:10.18632/aging.101611)
Supplement: Supplementary Figure [file aging-10-101611-s001.pdf]

SUPPLEMENTARY FIGURE

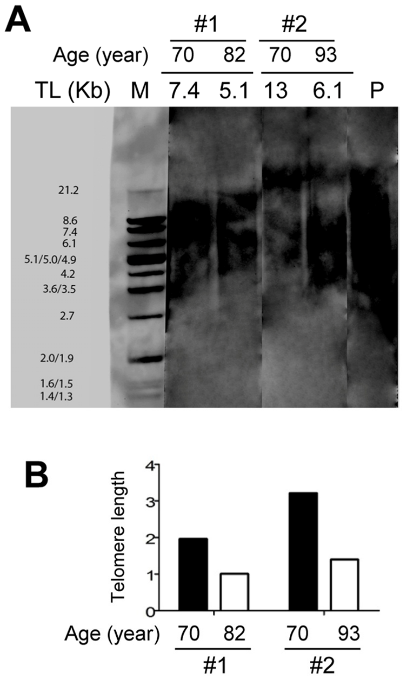

Supplementary Figure 1. DNA was derived from two individuals. Blood samples used were collected at ages 70 and 82 for the first individual, and at 70 and 93 for the second individual. (**A**) Southern blot assay was performed using a TeloTAGGG™ Telomere Length Assay kit. M: Molecular markers (kbs); P: Positive control. TL was quantified based on molecular markers. (**B**) qPCR quantification of relative TL from the same blood samples. Of note, the second individual displayed two separate TL signals, which indicates the presence of two cell populations with different TL in the blood.
